# Associations of self-reported atopic dermatitis with comorbid conditions in adults: a population-based cross-sectional study

**DOI:** 10.1186/s12895-020-00117-8

**Published:** 2020-12-17

**Authors:** Jevgenija Smirnova, Scott Montgomery, Magnus Lindberg, Åke Svensson, Laura von Kobyletzki

**Affiliations:** 1 School of Medical Sciences, Örebro University, Campus USÖ, S-701 82 Örebro, Sweden; 2 Department of Dermatology, Karlstad Central Hospital, Karlstad, Sweden; 3 Clinical Epidemiology Division, Department of Medicine, Karolinska Institute, Stockholm, Sweden; 4 Department of Epidemiology and Public Health, University College London, London, United Kingdom; 5 Department of Dermatology, Örebro University Hospital, Örebro, Sweden; 6 School of Medical Sciences, Lund University, Malmö, Sweden; 7 Department of Occupational and Environmental Dermatology, Skåne University Hospital, Lund University, Malmö, Sweden

**Keywords:** Atopic dermatitis, epidemiology, comorbidity, oral health, diabetes mellitus, hypertension, obesity

## Abstract

**Background:**

The objective of this study was to investigate the relationships between atopic dermatitis (AD) and other common chronic health conditions in adults.

**Methods:**

A cross-sectional survey was sent to a randomly selected population sample of 78,004 adults in Sweden. The questionnaires included measures of self-reported physical and mental health. Binary and multinomial logistic regression were used to examine the associations of AD with common chronic health conditions and psychological wellbeing.

**Results:**

AD was self-reported by 4,175 respondents, representing almost 14% of the study population of 34,313 adults. Our results showed positive associations between AD and chronic health disorders, including conditions of the oral cavity: chronic obstructive pulmonary disease (adjusted odds ratio [aOR] = 1.58, 95% confidence interval [CI]: 1.30 to 1.92), asthma (aOR = 2.13, 95% CI: 1.91 to 2.38), mild recurrent gastrointestinal symptoms (adjusted relative risk ratio [aRRR] = 1.78, 95% CI: 1.64 to 1.92), high blood pressure (aOR = 1.16, 95% CI: 1.06 to 1.26), obesity (aOR = 1.34, 95% CI: 1.23 to 1.47), mild joint pain (aRRR = 1.47, 95% CI: 1.35 to 1.61), mild headache or migraine (aRRR = 1.50, 95% CI: 1.38 to 1.64), caries (aOR = 1.25, 95% CI: 1.04 to 1.49), bleeding gums (aOR = 1.69, 95% CI: 1.38 to 2.08), periodontitis (aOR = 1.42, 95% CI: 1.13 to 1.77), sensitive teeth (aOR = 1.57, 95% CI: 1.35 to 1.82), and dry mouth (aOR = 1.52, 95% CI: 1.33 to 1.74). Adjustment for asthma and depression attenuated the magnitude of the associations between AD and the study outcomes. AD was also associated with poorer general psychological wellbeing.

**Conclusions:**

Adults reporting AD may be at increased risk of chronic disorders and decreased psychological wellbeing. Physicians should recognize that individuals with severe AD and those with comorbid asthma or depression may be especially vulnerable.

## Background

Atopic dermatitis (AD) is a chronic inflammatory skin disease common in children and adults, with a lifetime prevalence of about 10–20% [1]. This itchy skin disease, which may have a negative impact on the quality of life of people with the disease and their families, accounts for the largest global health burden caused by a skin disease [2, 3]. Health and quality of life may be especially impaired for individuals with concomitant mental health and atopic conditions [3, 4]. There is increasing evidence of the co-occurrence of common chronic diseases in individuals with AD [5].

Previous studies have reported associations between AD and both rheumatoid arthritis and inflammatory bowel disease [6]. Asthma has also been linked to AD, but, to the best of our knowledge, no studies have assessed the possible association between AD and chronic obstructive pulmonary disease (COPD), which also affects the epithelial tissues of the airways. Further, smoking has been reported to be associated with both AD and COPD, suggesting that it may be a shared risk factor for the two diseases. Co-occurrence of migraine has also been observed in individuals with AD [7, 8].

There are conflicting reports regarding the associations of AD with cardiovascular and metabolic disorders. In a recent meta-analysis, no statistically significant associations were found between AD and either hypertension or type 2 diabetes [9]. However, the studies included in the meta-analysis defined AD in various ways, and most included no information on the severity of AD [9]. Some previous observations of associations of AD with hypertension and type 2 diabetes may be explained by shared risk factors such as smoking and obesity [9–11].

Inflammatory diseases frequently involve conditions of the oral cavity, but few studies have investigated possible associations of AD with disorders of the oral cavity [12]. One national cross-sectional study conducted in the United States showed that children with severe AD had a higher prevalence of both toothache and bleeding gums [13]. To the best of our knowledge, no studies of adults with AD have examined the possibility of impaired health of the oral cavity.

The objective of this study was to examine the associations between self-reported AD and common chronic health conditions in adults sampled from the general population in Sweden.

## Methods

This study used data from Life and Health (Liv och Hälsa), a population survey conducted from March to June 2017 in Sweden. A postal questionnaire was distributed to a randomly selected population-based sample of 78,004 adults in five Swedish counties (Södermanland, Uppsala, Värmland, Västmanland, and Örebro), stratified by sex, age, and municipality. The questionnaire covered many topics, including health conditions, living habits, socioeconomic characteristics such as level of education, and questions derived from the 5-item World Health Organization Well-Being Index (WHO-5). The questionnaire included core questions for all study participants and additional questions specifically for the age groups of 18–29 years, 30–69 years, and 70 years and older. The response rate was 44%. Non-responders tended to be younger, men, people with lower levels of education, and individuals born in non-Nordic countries. The Life and Health study, including the methods of data collection and non-response analysis, have been described elsewhere [14]. The questionnaires used in the study are available on the municipality website [15].

Written informed consent was obtained from all participants. Ethical approval for this study was obtained from the Uppsala Regional Ethics Committee (registration number: 2015/417).

### Variables

The study measures were self-reported, and they were assessed using validated questions, whenever possible, or questions that have been used repeatedly in national and international surveys. The question used to assess AD was: ‘Do you have any of the following conditions?’, with ‘eczema’ as one of the listed conditions. This item had three response options: *no*; *yes, mild symptoms*; and *yes, severe symptoms*. Through discussion, a group of physicians, epidemiologists, and laypeople judged the face and content validity of this item as high. The AD question was also tested with 14 individuals with and without AD. The term ‘eczema’ was considered to be understandable by the general population in Sweden.

The health outcome variables used in this study were measured using the following question stem: ‘Do you have the following physician-diagnosed conditions?’. For the listed conditions of ‘asthma’, ‘COPD’, ‘high blood pressure’, ‘type 2 diabetes’, and ‘depression’, respondents chose between the responses of *yes* and *no*. For ‘gastrointestinal symptoms’, ‘joint pain’, and ‘headache or migraine’, three response options were: *no disease*; *yes, mild symptoms*; and *yes, severe symptoms*. Joint pain was defined as pain in one or several of the following locations: shoulders, neck, back, arms, elbows, legs, and knees. The items for COPD, high blood pressure, and type 2 diabetes were not included in the questionnaire for those aged under 30 years. Obesity was defined as a body mass index (BMI) of 30 or greater, calculated using questionnaire-reported weight and height. General subjective psychological wellbeing was estimated using the WHO-5 questions, and scores over 75 were interpreted as very good psychological wellbeing.

A detailed battery of questions on conditions of the oral cavity was included in the questionnaire for those aged 70 years and over, with items on caries, bleeding gums, periodontitis, sensitive teeth, and dry mouth. These conditions of the oral cavity were assessed using the question stem ‘Do you have any of the following conditions?’, with a binary answer option of *yes* or *no* for each condition.

Characteristics included in this study as potential confounding factors were sex, age group (18–29, 30–69, and ≥ 70 years), and smoking (*no*; *yes, daily or occasionally*). Respondents’ level of education (compulsory schooling, secondary, or higher education) was assessed using linked data from Statistics Sweden.

### Statistical analysis

Cross-tabulation was used to describe the study population. The associations between AD and the study outcomes were examined using binary logistic regression for the outcomes with two response categories (*no*, *yes*) and multinomial logistic regression for the outcomes with three response categories (*no*; *yes, mild symptoms*; *yes, severe symptoms*). Binary logistic regression produces odds ratios (ORs), whereas multinomial logistic regression compares each outcome category with a reference category and produces a relative risk ratio (RRR). All regression models were adjusted for sex, age group, smoking, and education level. The models for the associations of AD with conditions of the oral cavity were additionally adjusted for self-reported presence of permanent teeth. We re-estimated the models with additional adjustment for self-reported physician-diagnosed depression to evaluate the possible confounding effects of comorbid depression in individuals with AD.

We also assessed if the associations between the study outcomes and AD varied by presence of comorbid asthma. A new variable was created with three values: respondents who did not reported AD; respondents who reported having AD but not asthma; respondents who reported having both AD and asthma. Logistic regression analyses for associations between the study outcomes and the new variable were adjusted as described for the main analyses.

The association of AD with very good psychological wellbeing was estimated using logistic regression. The odds of having very good psychological wellbeing was assessed for mild and severe AD, and we compared these odds with the same odds for a comparison population of respondents who reported having diabetes, following an approach that has been used in a previous study [16].

The Bonferroni correction was applied to correct for multiple testing. Stratification by age and sex was performed.

Participants with missing values for the adjustment variables (sex, age group, smoking, and education level) were excluded from the analysis. A *p*-value lower than 0.05, or a 95% confidence interval (CI) that did not include 1.00, was used to indicate statistical significance. The analysis was conducted using SPSS version 26 statistical software (IBM Corp., 2019).

## Results

The study population consisted of 34,313 respondents (Table 1), 33,516 (43% of the target population) of that had complete questionnaire data after excluding those with missing data for the adjustment variables. There was a slight but statistically significant predominance of women, who comprised 54% of the sample. The median age of the respondents was 62 years. The highest level of educational attainment was compulsory schooling for 25% of the study population, secondary education for 43%, and higher education for 32%. AD was reported by 4,175 respondents (13.9% of the study population), including 419 respondents who reported severe AD. AD was most common in women aged 18–30 years. In this age group, 15.5% reported mild AD, and 3.5% reported severe AD.

**Table 1:** Population characteristics by atopic dermatitis diagnosis for 34 313 adults (including missing data)

	Total number in category (% of all in the study)	Atopic dermatitis		
		No 25 955 (86.1) n (%)	Yes, mild 3 756 (12.5) n (%)	Yes, severe 419 (1.4) n (%)
Sex				
Male	15 880 (46.3)	12 033 (46.4)	1 660 (44.2)	151 (36.0)
Female	18 433 (53.7)	13 922 (53.6)	2 096 (55.8)	268 (64.0)
Age				
18–29	5 393 (15.7)	3 991 (15.4)	623 (16.6)	133 (31.7)
30–69	15 769 (46.0)	11 921 (45.9)	1 880 (50.1)	175 (41.8)
70+	13 151 (38.3)	10 043 (38.7)	1 253 (33.4)	111 (26.5)
**Comorbid conditions**				
Chronic obstructive lung disease^a^				
No	22 772 (96.1)	19 217 (96.6)	2 690 (95.2)	226 (91.5)
Yes	912 (3.9)	674 (3.4)	135 (4.8)	21 (8.5)
Asthma				
No	26 163 (91.7)	22 190 (92.9)	2 993 (86.0)	295 (77.8)
Yes	2 368 (8.3)	1 691 (7.1)	486 (14.0)	84 (22.2)
Recurrent gastrointestinal symptoms				
No	22 453 (73.9)	19 680 (76.2)	2 391 (64.6)	205 (50.4)
Yes, mild	6 647 (21.9)	5 208 (20.2)	1 105 (29.9)	131 (32.2)
Yes, severe	1 267 (4.2)	936 (3.6)	205 (5.5)	71 (17.4)
High blood pressure^a^				
No	16 893 (66.1)	14 245 (67.2)	1 984 (66.0)	152 (56.9)
Yes	8 648 (33.9)	6 946 (32.8)	1 020 (34.0)	115 (43.1)
Diabetes type 2^a^				
No	21 844 (90.2)	18 398 (90.7)	2 604 (90.5)	213 (83.9)
Yes	2 383 (9.8)	1 876 (9.3)	274 (9.5)	41 (16.1)
Obesity (BMI > = 30)				
No	26 764 (81.8)	20 546 (82.5)	2 818 (78.0)	278 (71.1)
Yes	5 970 (18.2)	4 351 (17.5)	795 (22.0)	113 (28.9)
Joint pain^b^				
No	8 185 (26.9)	7 291 (28.5)	776 (21.1)	49 (11.9)
Yes, mild	17 539 (57.7)	14 696 (57.4)	2 228 (60.5)	225 (54.5)
Yes, severe	4 677 (15.4)	3 638 (14.2)	681 (18.5)	139 (33.7)
Headache or Migraine				
No	23 661 (78.7)	20 651 (80.3)	2 619 (71.7)	244 (60.2)
Yes, mild	5 547 (18.5)	4 432 (17.2)	881 (24.1)	122 (30.1)
Yes, severe	846 (2.8)	634 (2.5)	153 (4.2)	39 (9.6)
**Health**				
WHO-5 Well-being Index				
Very Good (WHO5 > 75)	14 804 (48.5)	12 817 (50.2)	1 483 (40.2)	84 (20.7)
Not Very Good	15 750 (51.5)	12 699 (49.8)	2 204 (59.8)	322 (79.3)
How would you rate your dental health?				
Very Good	10 388 (30.6)	8 292 (32.2)	985 (26.4)	101 (31.3)
Good	15 349 (45.2)	11 658 (45.2)	1 701 (45.6)	156 (37.4)
Not Good not Bad	5 404 (15.9)	3 910 (15.2)	672 (18.0)	86 (20.6)
Bad	2 113 (6.2)	1 464 (5.7)	295 (7.9)	45 (10.8)
Very Bad	687 (2.0)	450 (1.7)	77 (2.1)	29 (7.0)
**Social and Background Factors**				
Smoking				
No	30 111 (88.9)	23 022 (89.4)	3 298 (88.5)	335 (80.5)
Yes (daily or occasionally)	3 777 (11.1)	2 724 (10.6)	427 (11.5)	81 (19.5)
Highest level of education				
Compulsory schooling	8 343 (24.6)	6 090 (23.5)	785 (21.1)	119 (28.8)
Secondary education	14 688 (43.3)	11 076 (43.1)	1 651 (44.4)	195 (47.2)
Higher education	10 892 (32.1)	8 527 (33.2)	1 281 (34.5)	99 (24.0)

^a^Only age group over 30 years old

^b^Pain in one or several of the following: shoulders, neck, back, arms, elbows, legs, knees

### Chronic health conditions

The self-reported prevalence of common chronic health conditions and their distributions in the study population with no AD, mild AD, and severe AD are presented in Table 1. All chronic health conditions examined in the current study except type 2 diabetes were more prevalent in individuals with mild AD than in those without AD (Table 2). The association between type 2 diabetes and mild AD did not reach statistical significance (adjusted OR [aOR] = 1.11, 95% CI: 0.97 to 1.28). However, individuals with *severe* AD had statistically significantly higher odds of having type 2 diabetes (aOR = 1.96, 95% CI: 1.37 to 2.79) compared with those without AD.

**Table 2:** The associations between atopic dermatitis and study outcomes for 33 516 adolescents

	Mild atopic dermatitis		Severe atopic dermatitis	
	Unadjusted Model OR/RRR (CI 95%)	Adjusted Model aOR/aRRR^c^ (CI 95%)	Unadjusted Model OR/RRR (CI 95%)	Adjusted Model aOR/aRRR^c^ (CI 95%)
Chronic obstructive lung disease^a^				
No	Reference	Reference	Reference	Reference
Yes	1.45 (1.20 to 1.76)	1.58 (1.30 to 1.92)	2.74 (1.74 to 4.31)	2.84 (1.77 to 4.57)
Asthma				
No	Reference	Reference	Reference	Reference
Yes	2.16 (1.94 to 2.41)	2.13 (1.91 to 2.38)	3.87 (3.02 to 4.95)	3.54 (2.76 to 4.55)
Recurrent gastrointestinal symptoms				
No	Reference	Reference	Reference	Reference
Yes, mild	1.75 (1.62 to 1.90)	1.78 (1.64 to 1.92)	2.50 (2.00 to 3.12)	2.48 (1.98 to 3.11)
Yes, severe	1.85 (1.58 to 2.16)	1.85 (1.58 to 2.17)	7.38 (5.57 to 9.78)	6.33 (4.74 to 8.45)
High blood pressure^a^				
No	Reference	Reference	Reference	Reference
Yes	1.06 (0.98 to 1.15)	1.16 (1.06 to 1.26)	1.57 (1.23 to 2.01)	1.76 (1.36 to 2.29)
Type 2 diabetes^a^				
No	Reference	Reference	Reference	Reference
Yes	1.04 (0.91 to 1.19)	1.11 (0.97 to 1.28)	1.82 (1.29 to 2.57)	1.96 (1.37 to 2.79)
Obesity (BMI > = 30)				
No	Reference	Reference	Reference	Reference
Yes	1.34 (1.23 to 1.46)	1.34 (1.23 to 1.47)	1.90 (1.52 to 2.37)	2.01 (1.60 to 2.53)
Joint pain^b^				
No	Reference	Reference	Reference	Reference
Yes, mild	1.43 (1.31 to 1.56)	1.47 (1.35 to 1.61)	2.35 (1.71 to 3.23)	2.84 (2.05 to 3.93)
Yes, severe	1.78 (1.60 to 1.99)	1.91 (1.70 to 2.14)	6.01 (4.29 to 8.40)	7.57 (5.34 to 10.73)
Headache or Migraine				
No	Reference	Reference	Reference	Reference
Yes, mild	1.57 (1.44 to 1.71)	1.50 (1.38 to 1.64)	2.33 (1.87 to 2.91)	1.88 (1.49 to 2.37)
Yes, severe	1.92 (1.60 to 2.31)	1.80 (1.50 to 2.17)	5.39 (3.81 to 7.63)	3.89 (2.71 to 5.57)

The analyses used logistic regression for binary outcome variables and multinomial regression for categorical outcome variables with more than two categories

^a^Only age group over 30 years old

^b^Pain in one or several of the following: shoulders, neck, back, arms, elbows, legs, knees

^c^Adjusted for sex, age group, smoking and education level

A dose-dependent relationship between severity of health conditions and mild AD was seen: mild recurrent gastrointestinal symptoms (adjusted RRR [aRRR = 1.78, 95% CI: 1.64 to 1.92), severe recurrent gastrointestinal symptoms (aRRR = 1.85, 95% CI: 1.58 to 2.17), mild joint pain (aRRR = 1.47, 95% CI: 1.35 to 1.61), severe joint pain (aRRR = 1.91, 95% CI: 1.70 to 2.14), and mild headache or migraine (aRRR = 1.50, 95% CI: 1.38 to 1.64), severe headache or migraine (aRRR = 1.80, 95% CI: 1.50 to 2.17). The associations with severe AD showed higher magnitudes for all health conditions, compared with the associations with mild AD. Adjustment for age group, sex, smoking, and education level did not notably change these relationships, and applying the Bonferroni correction did not eliminate the statistical significance of these results. See Table 2 for detailed information about the relationships between common chronic health conditions and AD.

We re-estimated these associations after stratifying the sample by age and sex. The results were comparable to those of the main analysis, but some of the associations had wider CIs and borderline statistical significance. Adjustment for depression attenuated the magnitude of the associations between AD and chronic health conditions, but all the associations remained statistically significant.

The analyses of the AD subgroups with and without comorbid asthma showed that the majority of the examined associations were statistically significant for those who had AD but not asthma, however the magnitudes of these associations were lower for this group than for those with comorbid asthma. The exceptions were the associations of AD with COPD and with type 2 diabetes, which were statistically significant only in the AD subgroup with comorbid asthma.

### Dental comorbidity

Dental disorders were common in individuals with AD who were aged 70 years or older (Table 3). Mild AD was significantly associated with caries (aOR = 1.25, 95% CI: 1.04 to 1.49), bleeding gums (aOR = 1.69, 95% CI: 1.38 to 2.08), periodontitis (aOR = 1.42, 95% CI: 1.13 to 1.77), sensitive teeth (aOR = 1.57, 95% CI: 1.35 to 1.82), and dry mouth (aOR = 1.52, 95% CI: 1.33 to 1.74). The magnitudes of the associations between severe AD and these dental disorders were even higher, but only the relationships with bleeding gums (aOR = 2.35, 95% CI: 1.27 to 4.36) and dry mouth (aOR = 2.06, 95% CI: 1.34 to 3.17) were statistically significant. Adjustment for dry mouth slightly reduced the magnitude of all the examined associations between other dental disorders and AD. After additional adjustment for depression, the relationships between AD and the examined conditions of the oral cavity were attenuated but remained statistically significant except for the relationship between AD and periodontitis, which became non-significant. After applying the Bonferroni correction associations remained statistically significant, except the *p*-value for the association between caries and mild AD which changed from 0.015 to 0.075.

**Table 3:** Dental health and association with atopic dermatitis in 10 954 people over 70 years old

		Atopic dermatitis			Mild atopic dermatitis		Severe atopic dermatitis	
	Total number in category (% of all in the study)	No 10 043 (88.0) n (%)	Yes, mild 1 253 (11.0) n (%)	Yes, severe 111 (1.0) n (%)	Unadjusted Model OR (CI 95%)	Adjusted Model aOR^a^ (CI 95%)	Unadjusted Model OR (CI 95%)	Adjusted Model aOR^a^ (CI 95%)
Caries								
No	10 095 (86.6)	8 425 (87.2)	1 002 (84.4)	77 (81.9)	Reference	Reference	Reference	Reference
Yes	1 559 (13.4)	1 233 (12.8)	185 (15.6)	17 (18.1)	1.25 (1.05 to 1.50)	1.25 (1.04 to 1.49)	1.52 (0.94 to 2.79)	1.72 (1.00 to 2.97)
Bleeding gums								
No	10 686 (92.3)	8 955 (93.0)	1 054 (88.8)	82 (85.4)	Reference	Reference	Reference	Reference
Yes	890 (7.7)	677 (7.0)	133 (11.2)	14 (14.6)	1.68 (1.37 to 2.07)	1.69 (1.38 to 2.08)	2.20 (1.19 to 4.06)	2.35 (1.27 to 4.36)
Periodontitis								
No	10 622 (92.4)	8 895 (93.0)	1 062 (90.2)	84 (86.6)	Reference	Reference	Reference	Reference
Yes	871 (7.6)	673 (7.0)	115 (9.8)	13 (13.4)	1.44 (1.15 to 1.80)	1.42 (1.13 to 1.77)	1.47 (0.71 to 3.06)	1.41 (0.67 to 2.94)
Sensitive teeth								
No	9 354 (80.8)	7 854 (81.6)	892 (75.2)	76 (80.9)	Reference	Reference	Reference	Reference
Yes	2 218 (19.2)	1 766 (18.4)	294 (24.8)	18 (19.1)	1.51 (1.31 to 1.75)	1.57 (1.35 to 1.82)	1.19 (0.71 to 2.00)	1.27 (0.75 to 2.16)
Dry mouth								
No	8 141 (69.0)	6 868 (70.6)	746 (62.0)	48 (47.5)	Reference	Reference	Reference	Reference
Yes	3 661 (31.0)	2 865 (29.4)	458 (38.0)	53 (52.5)	1.46 (1.29 to 1.67)	1.52 (1.33 to 1.74)	2.20 (1.44 to 3.36)	2.06 (1.34 to 3.17)

^a^Adjusted for sex, age group, smoking, education level and permanent teeth

### Psychological wellbeing

In the study population, individuals with AD had a lower odds of having very good psychological wellbeing than did those without AD. Both those with mild AD and those with severe AD had significantly lower odds of having very good psychological wellbeing, compared with people with diabetes, and this finding showed a dose-dependent relationship (Table 4). There was no difference between individuals with mild AD and those with diabetes after adjustment. Those with severe AD had a significantly lower odds of having very good psychological wellbeing than did those with diabetes, even after adjustment.

**Table 4:** Association with WHO5 (very good) comparing atopic dermatitis and diabetes

	Unadjusted		Adjusted	
	OR (95% CI)	*p*-value atopic dermatitis vs. diabetes	aOR^b^ (95% CI)	*p*-value atopic dermatitis vs. diabetes
	Reference	Reference	Reference	Reference
Diabetes^a^ (*n* = 2 542)				
Mild atopic dermatitis (*n* = 3 182)	0.84 (0.75 to 0.93)	0.001	1.08 (0.96 to 1.21)	0.181
Severe atopic dermatitis (*n* = 332)	0.32 (0.25 to 0.42)	< 0.001	0.48 (0.36 to 0.63)	< 0.001

^a^All types of diabetes, including those with diabetes and AD

^b^Adjusted for sex, age group, smoking and education level

## Discussion

This study demonstrated that adults in Sweden with AD have an increased prevalence of self-reported chronic health conditions such as COPD, asthma, recurrent gastrointestinal symptoms, high blood pressure, obesity, joint pain, and headache or migraine. The observed associations show dose-dependent relationships for both AD severity and the severity of the examined chronic health conditions. We did not observe a statistically significant association between mild AD and type 2 diabetes; however, those with severe AD had a statistically significantly higher prevalence of type 2 diabetes, compared with those without AD. To the best of our knowledge, this is the first report of a positive association between AD and conditions of the oral cavity, such as caries, bleeding gums, periodontitis, sensitive teeth, and dry mouth, among older adults. Our findings also showed that AD was associated with decreased general psychological wellbeing, with a magnitude that is at least as high as that for diabetes. The findings also demonstrated that AD is common in adults, affecting almost 14% of the adult population in Sweden.

Our study results are in line with previous studies suggesting increased susceptibility to immune-mediated diseases in individuals with AD [17, 18]. Schmitt et al. previously reported that individuals with AD had increased risks of rheumatoid arthritis and inflammatory bowel disease in a large cohort study in Germany [6]. Likewise, the current study found that individuals with AD have increased odds of joint pain and recurrent gastrointestinal symptoms. Several possible explanations for these findings have been suggested. For example, there may be shared genetic susceptibility loci between AD and autoimmune diseases [17, 19]. Another potential mechanism is related to prolonged systemic inflammation increasing an individual’s risk for developing several autoimmune diseases [5, 17]. Surveillance bias should be considered in studies investigating comorbidity patterns in individuals with chronic diseases because of the relatively higher health service use in this population.

In the current study, asthma was found to be associated with AD, as expected. Our study is the first to describe the association between COPD and AD. Immunoglobulin E (IgE) sensitization has previously been shown to be more frequent in patients with COPD than in the general population [20]. However, our analysis of AD subgroups with and without comorbid asthma did not confirm a significant association with COPD for those who have AD without asthma. It is likely that individuals without comorbid asthma had more mild AD and therefore did not show an association with COPD. Another possibility is misdiagnosis because of the similarity of asthma and COPD symptoms; people who reported COPD might have had asthma instead of COPD or both asthma and COPD.

Several previous studies have aimed to investigate the associations between cardiovascular diseases and AD, with conflicting results [5, 9]. These differences may be explained by differences in the study designs and diagnostic criteria used in previous studies [21]. Our study findings of relatively high odds of hypertension and type 2 diabetes in individuals with severe AD are in line with another recent Swedish study [22]. The present study also adds to existing information on the AD–obesity relationship in the Swedish population, especially for those with severe AD. Our findings on this topic are in line with a recent meta-analysis showing an association between AD and obesity in North America and Asia [11]. Among the participants in our study, individuals with severe AD were more frequently smokers than were those with mild AD or without AD. It is possible that individuals with severe AD have a higher prevalence of adverse cardiovascular events because of shared associated factors such as obesity, smoking, hypertension, and diabetes.

Migraine has previously been linked to allergic diseases such as asthma and allergic rhinitis, and some studies have reported an association with AD in children and adolescents as well [7, 8]. Our study adds new knowledge about the increased prevalence of migraine and headache in adults with AD. The pathophysiology of migraine is still unknown, but the roles of mast cell activation and increased levels of pro-inflammatory cytokines, which may be related to allergic inflammation, have been discussed [23].

Recent work has suggested the suspected involvement of the oral cavity in AD [24], and several studies have demonstrated poorer dental health in children with AD compared with those without AD [13, 25]. To our knowledge, our study is the first to investigate associations of AD with conditions of the oral cavity, such as caries, bleeding gums, periodontitis, sensitive teeth, and dry mouth, in the older adult population. The underlying mechanisms behind these associations need to be explored in future work. We speculate that the relatively high use of antibiotics and antihistamines in individuals with AD, may affect the microbiome of the oral cavity, inflammation, and dryness of the mouth, may be part of the mechanism for this association. A reduction in the magnitude of the associations of caries, bleeding gums, periodontitis, and sensitive teeth with AD after adjusting for dry mouth may indicate that dry mouth is part of the explanation for the relationship between AD and poor dental health.

The associations with psychological wellbeing are consistent with previous work, and our results showed a link between AD and decreased general psychological wellbeing, especially for those with severe AD [26]. Impaired sleep because of itching and adversely affected self-esteem because of visible disease symptoms may be part of the explanation for this relationship. AD in late adolescence has been found to be associated with antidepressant medication use in middle age, but the same association for asthma was not statistically significant after adjusting for covariates [27].

### Study strengths and limitations

A strength of our study is its use of a large, randomly selected population sample, which allowed us to perform analyses with AD subgroups and to investigate relatively uncommon outcomes. The study results consistently showed greater magnitude of associations with the study outcomes for severe AD than for mild AD, demonstrating dose-dependent relationships. These findings suggest that there may be causal relationships between AD and the examined outcomes. In addition, we were able to adjust for potentially important potential confounding factors such as smoking and education level and to evaluate the effects of comorbid asthma and depression on the study results. Further, we used the Bonferroni correction to correct for the possibility of chance statistically significant findings due to multiple testing. However, despite these statistical approaches, we cannot eliminate the possibility of residual confounding.

Another limitation of our study is the cross-sectional design, which does not allow for evaluation of causality. Our study also had a low response rate. Older people and highly educated people were slightly overrepresented in our study population, which may indicate selection bias. Although adjustment for education level was performed for all analyses, residual confounding because of selection bias cannot be excluded. The non-response analysis suggested that our study population is representative of the target population overall. The age-stratified analyses showed that the associations did not differ across age groups; thus, the slight overrepresentation of older adults should not have influenced the results. A previously published age-standardized report using the same dataset showed disease prevalence rates that were similar to those previously reported for the adult population in Sweden [14]. Because the data used in this study were self-reported, we cannot exclude the possibility of reporting bias, including recall bias. The data on recurrent gastrointestinal symptoms, joint pain, and headache may refer to several heterogeneous health conditions. The absence of a gold standard for questionnaire-based AD diagnosis and the fact that respondents may confuse AD with other skin conditions are challenges for questionnaire-based epidemiological studies [21]. A validation study conducted by Silverberg et al. concluded that a self-reported diagnosis of eczema based on a single question (‘Have you ever been told by a doctor or other health professional that you had eczema or any kind of skin allergy?’) had sufficient validity for the epidemiological study of AD [28]. Another study conducted among adults in Sweden reported that the self-reported lifetime prevalence of atopic dermatitis, assessed by the question ‘Have you had childhood eczema?’, was 13.7%, which is in line with our findings [29]. Additionally, despite adjusting for smoking in our study, we cannot rule out a residual confounding effect of exposure to smoking on the associations between AD and health conditions, particularly COPD and periodontitis [10].

## Conclusions

AD is associated with several common chronic health conditions including conditions of the oral cavity and with poor general psychological wellbeing in adults. Individuals with severe AD and those with comorbid asthma or depression may be especially vulnerable. These findings contribute to the existing information about comorbid conditions among people with AD. Future research should focus on disease mechanisms and optimizing treatment with regard to comorbidity.

## Data Availability

The data used in the study contain potentially identifying and sensitive patient information thus, cannot be made publicly available for legal restrictions arising from general data protection regulations and restrictions imposed for ethical approval (registration number: 2015/417). The corresponding author is not allowed to share the data on request. The data are not owned by our research group and a new ethical application would be required to request the data from those responsible.

## Abbreviations

AD: Atopic dermatitis
COPD: Chronic obstructive pulmonary disease
WHO-5: The 5-item World Health Organization Well-Being Index
OR: Odds ratio
aOR: Adjusted odds ratio
RRR: Relative risk ratio
aRRR: Adjusted relative risk ratio
CI: Confidence interval
BMI: Body mass index
